# Case Report: One case of pregnancy complicated by large cell neuroendocrine carcinoma of the cervix with syndrome of inappropriate secretion of antidiuretic hormone

**DOI:** 10.3389/fonc.2025.1648644

**Published:** 2025-11-28

**Authors:** Changhong Dong, Baoyu Zhu, Guoying Miao, Zhangcai Zheng

**Affiliations:** Department of Radiotherapy, Gansu Provincial Maternity and Child Health Care Hospital, Lanzhou, China

**Keywords:** LCNEC of the cervix, pregnancy, SIADH, hyponatremia, tolvaptan

## Abstract

This article reports an extremely rare case of a 31-year-old pregnant woman diagnosed with large cell neuroendocrine carcinoma (LCNEC) of the cervix complicated by syndrome of inappropriate antidiuretic hormone secretion (SIADH). Admitted at 35^+^¹ weeks gestation due to vaginal bleeding, she was diagnosed with cervical LCNEC and pelvic lymph node metastasis. Following cesarean delivery, she developed severe hyponatremia (as low as 92 mmol/L) leading to coma during chemotherapy, meeting the criteria for SIADH. The hyponatremia was successfully corrected with the selective vasopressin V2 receptor antagonist tolvaptan. The patient subsequently achieved complete remission (CR) after concurrent chemoradiotherapy. However, the disease recurred with multiple metastases six months later. Despite multiple lines of therapy, she succumbed to multiple organ failure 19 months after initial diagnosis. This case highlights the highly aggressive nature and poor prognosis of LCNEC complicated by SIADH during pregnancy. Tolvaptan proved effective for the associated refractory hyponatremia but required careful monitoring to avoid sodium overcorrection. Dynamic serum sodium monitoring may serve as a potential biomarker for tumor recurrence. A review identified deficiencies in the management, including initial insufficient investigation into the cause of hyponatremia, aggressive fluid therapy exacerbating the condition, and delays in multidisciplinary collaboration and systemic therapy. This case underscores the critical importance of multidisciplinary collaboration and early, aggressive systemic treatment in managing such complex and rare malignancies.

## Introduction

Cervical cancer in pregnancy refers to cancer cases diagnosed during pregnancy, as well as those diagnosed 6 – 12 months postpartum. The incidence of cervical cancer is low, accounting for approximately 1 – 3% of all pregnancies (1). Pregnancy may accelerate cancer progression, and some researchers have found that estrogen, progesterone, and human chorionic gonadotropin levels during pregnancy are positively correlated with HPV(human papillomavirus)16 and HPV18 infections, which suggests that pregnancy may promote cervical cancer progression. Primary cervical neuroendocrine carcinoma accounts for less than 2% of cervical malignancies, among which small-cell neuroendocrine carcinoma is relatively common. On the other hand, LCNEC(large-cell neuroendocrine carcinoma) is very rare, accounting for less than 0.2% of cervical malignancies (2).The colposcopic cervix may present as a raised mass, polypoid growth, erosive bleeding focus, or an endogenous diffuse infiltration in a barrel shape, similar to cervical squamous cell carcinoma or adenocarcinoma. However, cervical LCNEC is a high-grade cancer composed of large cells with neuroendocrine differentiation. Its biological behavior and clinical course are different from those of cervical squamous cell carcinoma or adenocarcinoma. It usually develops vascular and lymphatic metastasis early during its course, progresses rapidly, and is prone to recurrence with distant metastasis. Additionally, it has a high mortality rate, and the overall median survival time is less than 24 months (3). Immunohistochemical analysis has revealed neuroendocrine features in some LCNEC cases; however, their clinical presentations are mostly akin to those of common cervical malignancies. These manifestations often include abnormal vaginal bleeding, vaginal discharge, cervical vegetation, pain, or even solely cytological abnormalities. Furthermore, only a minority of LCNEC patients exhibit symptoms of neuroendocrine tumors, such as SIADH (syndrome of inappropriate antidiuretic hormone secretion). This study outlines a case involving a pregnant patient diagnosed with LCNEC accompanied by SIADH at our hospital.

## Case description

**Figure 1:**
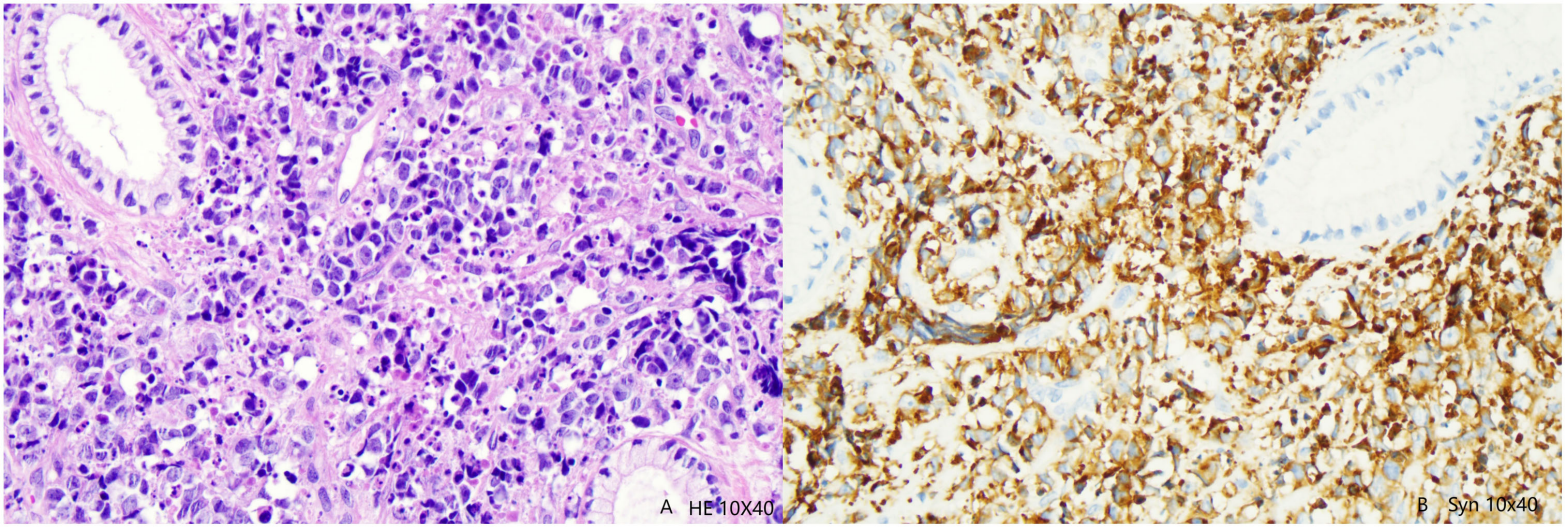
**(A)** Tumor cells are mainly in the form of nests, flaks and cribs; tumor cells diffused into the cervical stroma and invaded the cervical mucosal epithelium. HE high magnification(10×40). **(B)** The tumor cells were synaptophysin positive.

A 31-year-old female patient arrived at the hospital on September 9, 2021, presenting with 35 + 1 weeks of gestation and intermittent vaginal bleeding for two weeks. Admission Physical Examination: Normal vulvar development, unobstructed vagina. Cervical structure absent. A cauliflower-like mass approximately 7 cm in diameter palpable on the surface. Negative for bleeding on palpation. Vaginal vault and upper third of vagina involved. Fundus 3 fingers below xiphoid process. Tripartite examination: Palpation of bilateral parametrial spaces unsatisfactory. Rectal mucosa smooth. No bleeding upon withdrawal of finger. Gynecological ultrasound results revealed: 1. intrauterine singleton pregnancy, and 2. cervical mass lesions. TCT (ThinPrep cytologic test) indicated AGC-NOS (atypical glandular cells), and HPV typing showed HPV 18 positivity. Colposcopy examination suggested cervical cancer, as the cervix appeared hard, was irregularly enlarged to approximately 6-7 cm in diameter, with brittle surface tissue and atypical blood vessels. Immunohistochemical findings included P16 (+), CK5/6 (-), Ki67 (+ 80%), P53 (wild-type), CAM5.2 (-), CKL (-), Pax2 (-), CgA (-), and Syn (+). Pathological diagnosis was determined as invasive carcinoma of the cervix; immunohistochemical findings were consistent with neuroendocrine carcinoma, and showed a propensity for large cell neuroendocrine carcinoma (see **Figures 1A, B**).

**Figure 2:**
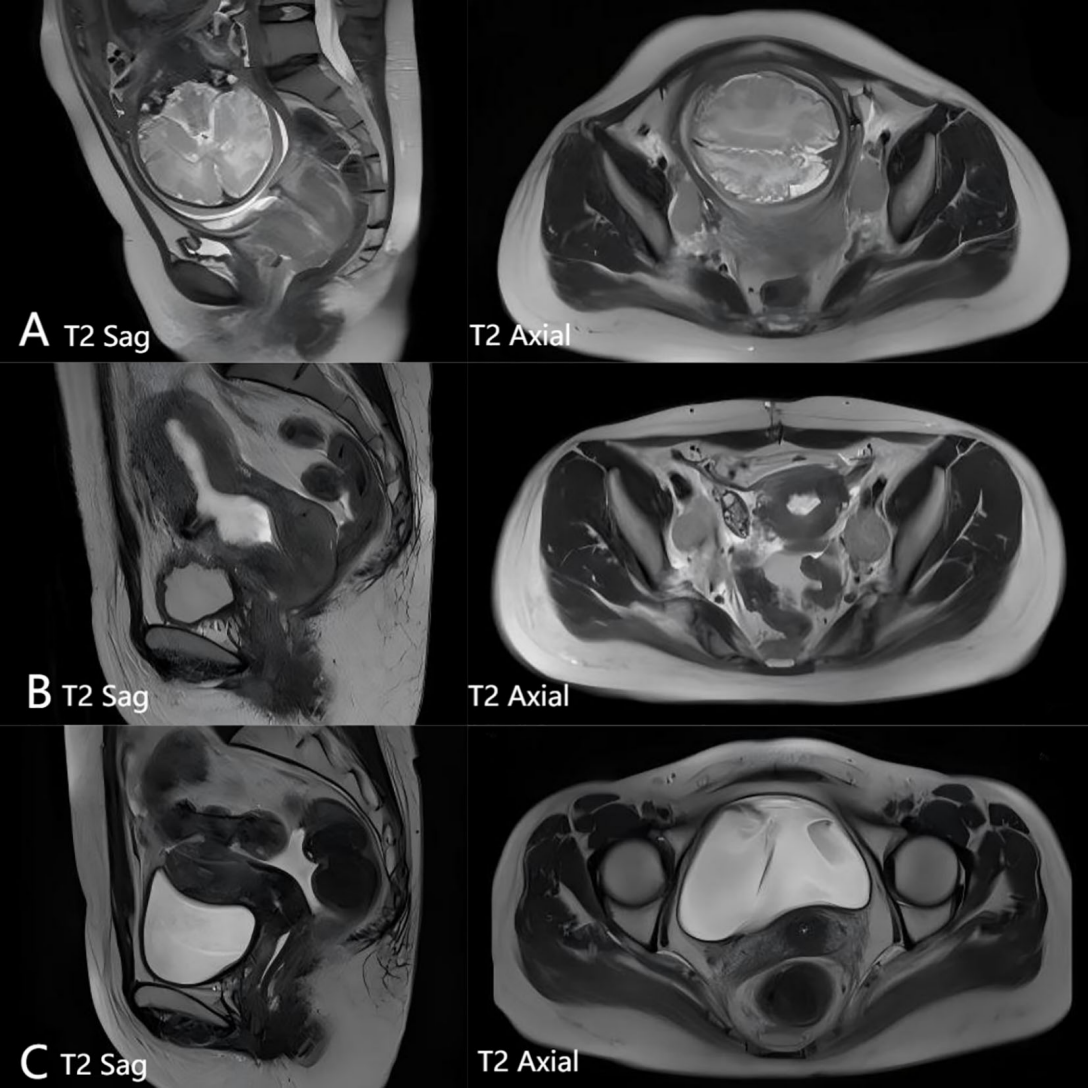
**(A)** Pelvic magnetic examination before treatment: Cervical mass with maximum transverse dimension of 7.9 cm × 5.6 cm and longitudinal dimension of 4.2 cm. Bilateral parametrial fat planes involved. Vaginal vault involved. No significant involvement of bladder or rectum noted. Multiple enlarged lymph nodes were noted bilaterally along the internal and external iliac vessels, with the largest located on the left side, measuring approximately 3.4 cm × 2.5 cm. Single intrauterine pregnancy, cephalic presentation, with the umbilical cord wrapped twice around the neck. **(B)** 2 PE cheng solution after chemotherapy pelvic MRI examination: Cervical mass significantly reduced in size compared to previous imaging, with maximum transverse dimension of 4.1 cm × 3.4 cm and longest diameter of 3.7 cm. Bilateral parametrial fat spaces involved, vaginal vault involved. No significant involvement of bladder or rectum noted. Multiple enlarged lymph nodes were observed along the internal and external iliac vessels bilaterally. The largest node, located on the left side, measured approximately 2.7 cm × 2.8 cm × 4.8 cm and showed reduction in size compared to previous findings. **(C)** Pelvic MRI check following treatment: Cervical lesion has almost disappeared.

Pelvic MRI, September 9, 2021: Cervical mass with maximum transverse dimension of 7.9 cm × 5.6 cm and longitudinal dimension of 4.2 cm. Bilateral parametrial fat planes involved. Vaginal vault involved. No significant involvement of bladder or rectum noted. Multiple enlarged lymph nodes were noted bilaterally along the internal and external iliac vessels, with the largest located on the left side, measuring approximately 3.4 cm × 2.5 cm. Single intrauterine pregnancy, cephalic presentation, with the umbilical cord wrapped twice around the neck(see **Figure 2A**). Therefore, the patient was finally diagnosed with cervical malignancies (large cell neuroendocrine carcinoma IIIC1r FIGO(International Federation of Gynecology and Obstetrics)2018).

On September 10, 2021, the patient underwent a lower uterine segment cesarean section under combined spinal-epidural anesthesia, resulting in the delivery of a healthy male baby. Post-delivery, the patient was diagnosed with cervical large cell neuroendocrine carcinoma, classified as locally advanced cervical cancer with multiple pelvic lymph node metastases. Considering the highly malignant nature of the tumor and the patient’s need for recovery following surgery, a PE regimen (cisplatin 100 mg D1 + etoposide 150 mg, D1-3) was initiated on September 18, 2021. However, on the third day of chemotherapy, the patient suddenly became unconscious, experiencing foaming at the mouth, limb twitching, and trismus.

Physical examination revealed bilateral anisocoria, weak light reflex, heightened muscle tone in all four limbs, and bilateral positive Babinski signs. Blood gas analysis indicated metabolic acidosis. Brain CT (computed tomography) scans showed no significant abnormalities. The patient’s symptoms improved following symptomatic treatments such as sedation, analgesia, intracranial pressure reduction, acidosis correction, and sodium supplementation. Nonetheless, serum sodium levels fluctuated between 92 and 131 mmol/L.

On October 12, 2021, the patient received two courses of PE chemotherapy, accompanied by daily intravenous infusions of 3% concentrated sodium chloride (25-30g) and oral sodium salt (6g) to correct hyponatremia, and to maintain sodium ion levels at 120-130 mmol/L. Endocrinologists were consulted and determined the patient’s urine sodium level to be 332 mmol/24 hours (normal range: 130-260 mmol/24 hours), urine osmotic pressure at 400 mOsm/(kg·H2O), and plasma osmotic pressure at 264.0 mOsm/(kg·H2O). Thyroid ultrasound, adrenal CT, pituitary MRI, blood cortisol, ACTH, and thyroid function tests showed no significant abnormalities. As the patient had normal blood pressure and no recent diuretic use, SIADH caused by large cell neuroendocrine carcinoma was highly suspected.

**Figure 3:**
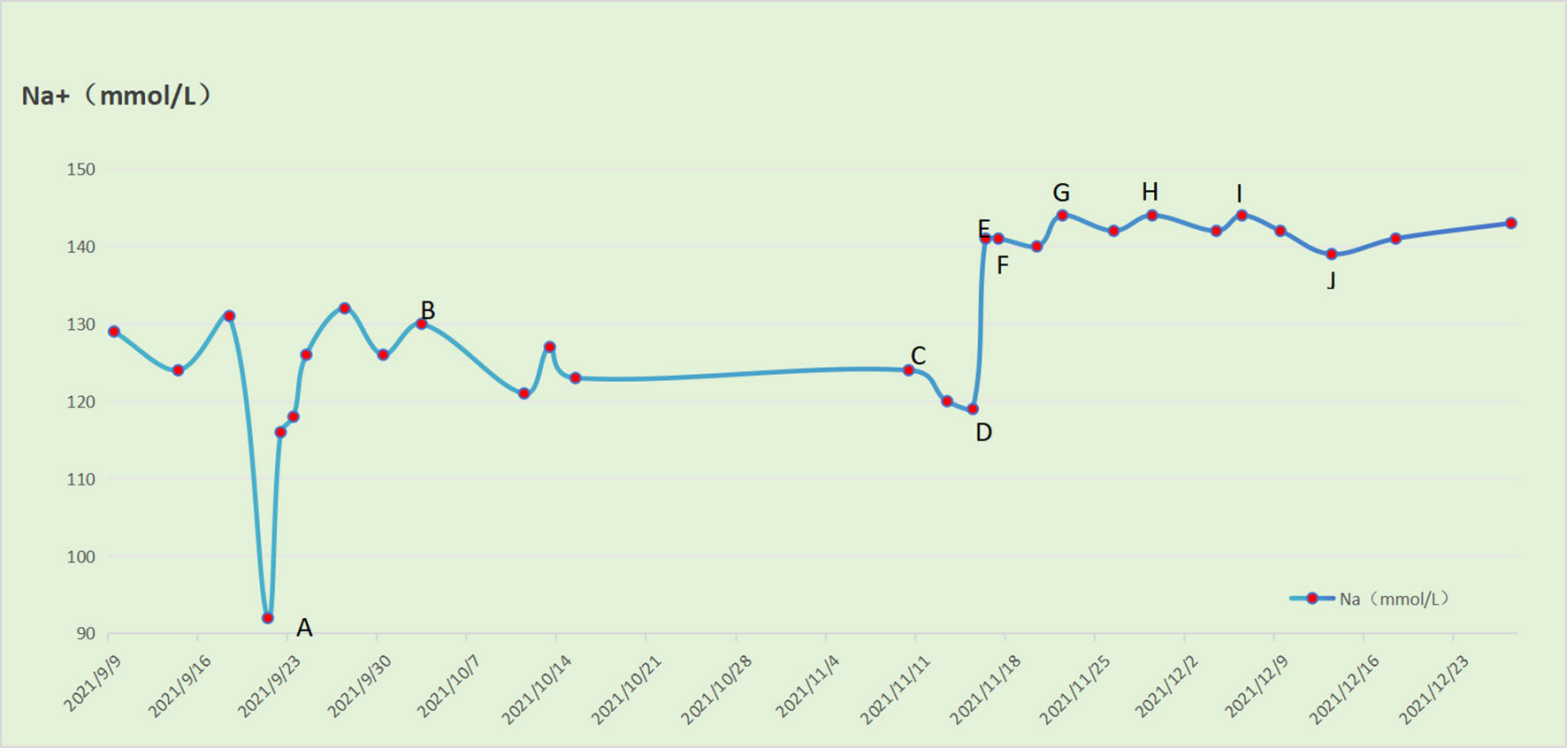
Relationship between gradual reduction of tolvaptan tablets and serum Na level in patients **(A)** The patient first developed severe hyponatremia (92 mmol/L) on day 3 after the 1st course of EP regimen chemotherapy, and received intravenous pumping of 3% NaCl 45-55 ml/h. **(B)** The patient received oral 10% NaCl 5 ml/times and 3 times/day and intravenous infusion of 3% NaCl 1000 ml/d. **(C)** The patient received oral 10% aCl 5 ml/time 3 times/day and intravenous infusion of 3% NaCl 1250 ml/d during the 3rd course of chemotherapy with EP regimen. **(D)** The patient received oral tolvaptan tablets 15 mg/day, 10% NaCl 5 ml/time 3 times/day and intravenous infusion of 3% NaCl 750 ml/d. **(E)** The patient received oral tolvaptan tablets 15 mg/day, 10% aCl 5 ml/time and 3 times/day. **(F)** The patient received oral tolvaptan tablets 15 mg/day. **(G)** The patient received oral tolvaptan tablets 11.25mg/day. **(H)** The patient received oral tolvaptan tablets 7.5 mg/day. **(I)** The patient received oral tolvaptan tablets 3.75 mg/day. **(J)** The patient discontinued oral tolvaptan tablets.

In response, tolvaptan tablets (15 mg/day) were administered to the patient, stabilizing blood sodium levels between 134 and 145 mmol/L. The dosage was gradually reduced by 3.75 mg weekly, and after four weeks, it was discontinued and replaced with oral sodium salt capsules (6 g/day). The patient’s blood sodium levels remained within the normal range (see **Figure 3**). Pelvic MRI following two courses of PE chemotherapy revealed a significant reduction in the cervical mass, with the maximum cross-sectional area measuring 4.1 cm x 3.4 cm, and the longest diameter at 3.7 cm. Multiple enlarged lymph node shadows were observed adjacent to the bilateral internal and external iliac vessels, the largest of which was located on the left side, measuring approximately 2.7 cm x 2.8 cm x 4.8 cm (see **Figure 2B**). Therefore, the tumor efficacy evaluation was PR (partial remission) (according to Recist 1.1 criteria).

After the third course of PE chemotherapy and stable blood ion levels, the patient transitioned to concurrent chemoradiotherapy using the VMAT(volumetric modulated arc therapy) technique. The target range for radical radiotherapy included: GTV (gross tumor volume) - imaging positive lymph nodes; CTV (clinical target volume) - cervix, uterine body, parametrium, upper two-thirds of the vagina, para-aortic drainage area, and pelvic lymph node drainage area. Radiotherapy doses were set at 56 Gy/28 fractions/6 weeks for PGTV(planning gross tumor volume) and 50.4 Gy/28 fractions/6 weeks for PTV(planning target volume). Four 3-dimensional intracavitary brachytherapy sessions (HRCTV(high-risk clinical target volume): 600 cGy/f, IRCTV(Intermediate-risk clinical target volume): 400 cGy/f) were administered following completion of external beam radiation.

During radiotherapy, the patient received one course of chemotherapy concurrent with the PE regimen, and two supplementary courses of PE regimen chemotherapy after completing radiotherapy. The patient reported no significant discomfort throughout the treatment, and sodium ion levels consistently remained within the normal range. A pelvic MRI reexamination after chemoradiotherapy completion showed that the cervical mass had essentially disappeared (see **Figure 2C**), and the therapeutic effect was evaluated and determined to be a CR (complete response). At the time of the 6-month follow-up, there have been no signs of recurrence.

**Figure 4:**
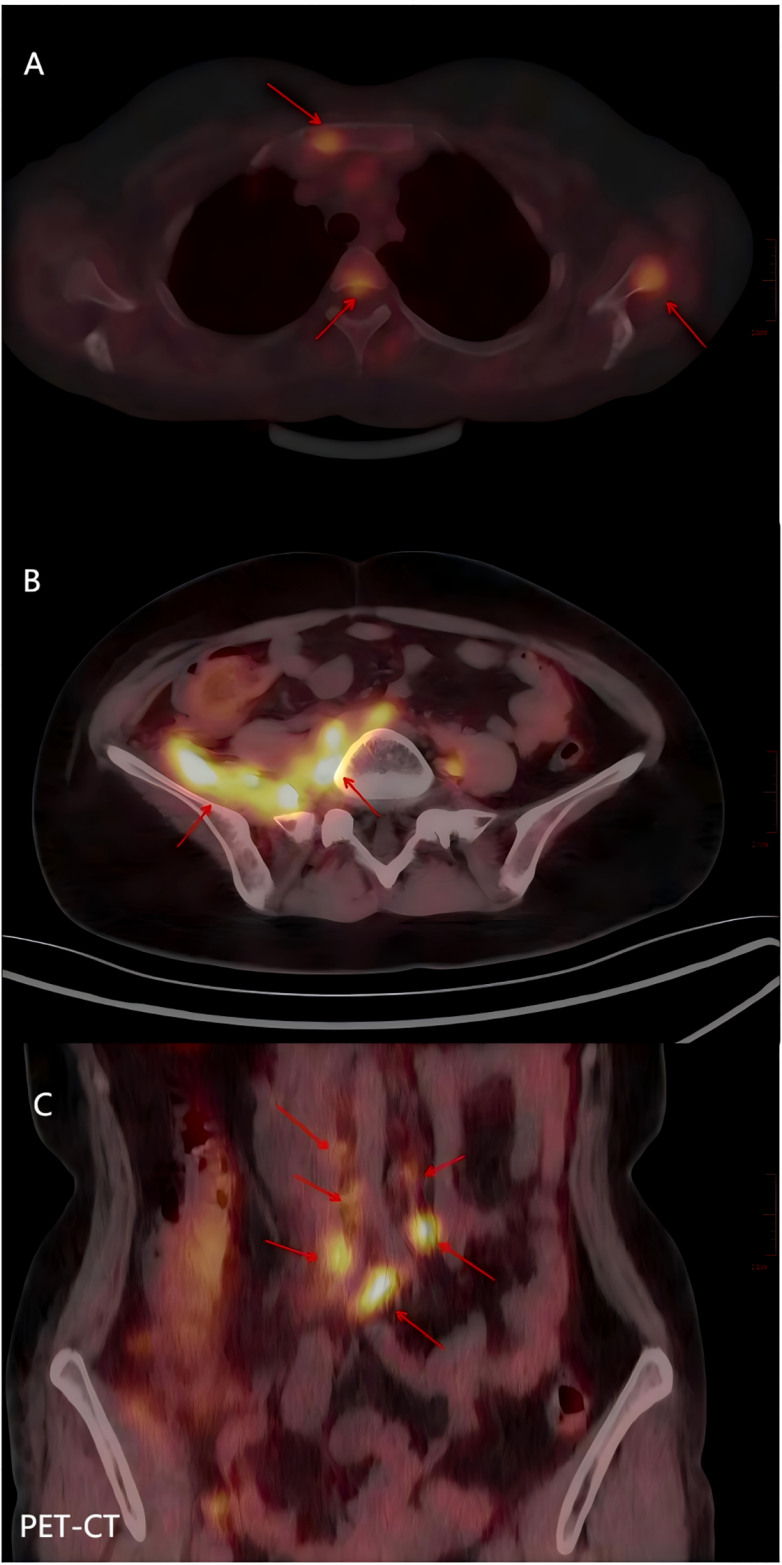
PET-CT of a patient who relapsed 8 months after completion of chemoradiotherapy **(A)** The sternum, thoracic vertebrae and scapula had increased FDG metabolism, and metastatic carcinoma was considered. **(B)** The FDG metabolism of the right pelvic wall muscle was increased, and metastatic carcinoma was considered. **(C)** The FDG metabolism of lymph nodes adjacent to bilateral common iliac vessels and abdominal main vessels was increased, and metastatic carcinoma was considered.

Follow-up: In August 2022, 6 months after radical chemoradiotherapy, the patient was re-examined and re-issued second-course radiotherapy to the right pelvic paraciliac vascular lymph nodes. Systemic treatment was initiated after a PET-CT scan on October 17, 2022, showed numerous lymph node metastases, bone metastases, and metastases to the right pelvic wall(see **Figure 4**).

The patient passed away in May 2023 due to multiple organ failure, with an overall survival (OS) of 19 months.

Limitations: At the first visit, not enough attention was paid to hyponatremia, and the patient was only given symptomatic treatment with sodium supplementation. Due to lack of experience in the treatment of the syndrome accompanied by improper secretion of antidiuretic hormone, severe hyponatremia and related complications occurred following the first chemotherapy.

## Discussion

### Management of cervical cancer associated with pregnancy

NENs(Neuroendocrine neoplasms) are aggressive malignancies originating from neuroendocrine cells. In humans, NENs are typically located in the gastrointestinal tract, the pancreas, and the lung, but in rare cases, they may also occur in other organs, such as the female reproductive tract. The cervix is the most common site, followed by the ovaries and the endometrium. Cervical LCNEC is a rare and aggressive tumor, accounting for approximately 1 – 1.5% of cervical cancers, and is often misdiagnosed with a dismal prognosis (4).

Cervical cancer in pregnancy refers to cases diagnosed during pregnancy, as well as those diagnosed 6 – 12 months postpartum. The incidence is low, accounting for approximately 1– 3% of all pregnancies (1). Pregnancy may accelerate cancer progression, and some researchers have found that estrogen, progesterone, and human chorionic gonadotropin levels during pregnancy are positively correlated with HPV16 and HPV18 infections, which suggests that pregnancy may promote cervical cancer progression. Factors such as increased lymphatic circulation and blood flow into the reproductive organs of pregnant women, decreased immunity during the first trimester, and postpartum cervical dilation may accelerate tumor metastasis, thereby promoting cervical cancer development.

Regarding the deficiencies in the treatment process of this patient: The clinicians lacked awareness of multidisciplinary collaboration. This case initially presented with multiple metastatic lymph nodes in the pelvis, the largest measuring 5.2 cm in diameter. The obstetricians could have collaborated with gynecological oncologists to remove the enlarged metastatic pelvic lymph nodes after the successful delivery of the fetus. This would have reduced the tumor burden for the subsequent radiotherapy, thereby decreasing the radiation dose to the surrounding normal tissues and ultimately mitigating the adverse effects experienced by the patient during radiotherapy.

### Treatment of cervical LCNEC

Patients with NECC(neuroendocrine cervical cancer) should start systemic therapy as soon as possible after termination of pregnancy, and the treatment regimen should be the same as that during non-pregnancy. The SGO(Society of Gynecologic Oncology) in 2011 and GCIG(Gynecologic Cancer Inter-Group) in 2014 recommended comprehensive therapy for NECC. For NECC patients diagnosed during middle and late pregnancy, pregnancy should be terminated by cesarean section shortly after diagnosis. In the case that we reported, pregnancy was terminated by cesarean section on the second day after the discovery of NECC. In pregnant women with cervical cancer consisting of large cervical tumors, cesarean section is the preferred method for delivering fetuses, as vaginal delivery carries risks of vaginal tears, massive bleeding from scar incisions, and tumor metastasis. When the tumor is locally advanced, transverse cesarean sections should be avoided due to the risk of cutting or tearing the tumor; classical vertical incisions can reduce bleeding and avoid damaging tumor vessels. Neuroendocrine tumors in pregnancy generally lead to recommendation for termination of pregnancy to provide radical treatment due to their invasiveness, tendency to metastasize distally, and their unknown origin. The placenta would be examined by pathology after operation to determine whether there was metastasis (5). However, maintenance of pregnancy was shown to be possible in certain common histological types of gynecological cancers. In one case, the patient experienced severe lower abdominal pain and vaginal bleeding, a large tumor was found, with and a maximum diameter of approximately 7.9 cm. The pregnant woman chose to undergo a lower uterine segment vertical incision cesarean section under combined spinal-epidural anesthesia at 35 + 1 weeks of gestation, resulting in the delivery of a healthy male baby. During the follow-up, the child was healthy and had no abnormal manifestations.

LCNEC is very rare, accounting for less than 0.2% of cervical malignancies. Because it is highly malignant, has rapid progression, poor prognosis, and other characteristics, clinical course and treatment for LCNEC are different from those of common cervical cancer types. Therefore, early detection and early treatment is very important. Regimens for patients with cervical LCNEC have long followed those for SCNEC(small cell neuroendocrine carcinoma). There is no uniform treatment for cervical SCNEC, and a meta-analysis of 3,538 cases of NECC showed that the most common initial treatment for NECC is radical surgery combined with chemotherapy (6). There is no standard chemotherapeutic regimen, but platinum combined with etoposide is the most commonly used treatment, and radiation therapy is also an option for patients with locally advanced disease. Comprehensive treatment, including surgery, chemotherapy, and radiotherapy, is recommended for cervical neuroendocrine tumors. Although it remains controversial, chemoradiotherapy is currently the treatment of choice for locally advanced NECC. The 2022 edition of the expert guidance opinion on the diagnosis and treatment of neuroendocrine carcinoma of the uterine cervix recommends that patients receive two cycles of neoadjuvant chemotherapy, with two to four supplemental cycles of chemotherapy after completing radiation therapy, to obtain a total of six chemotherapy cycles (7). Salvo et al. (8) reported that concurrent cisplatin and etoposide chemotherapy administered during radiotherapy is safe, and that cisplatin and etoposide chemotherapy could be administered after completing radiation therapy. In our case, the patient had locally advanced cervical cancer, lymph node metastasis, and was no longer a candidate for surgery. As a result, EP chemotherapy was administered after cesarean section, followed by radical concurrent chemoradiotherapy (EP regimen concurrent) more than one month after surgery. Two sequential two-course PE chemotherapy (a total of six courses) was also performed after completing radiation therapy. Although the tumor response was determined to be CR, cancer recurrence occurred only 6 months after the end of radiotherapy and chemotherapy, and the patient lost her life 10 months later.

Prodromidou et al. (3)reported that A total of 31 case studies including 87 LCNEC patients were identified. Median patients’ age was 41 years (range: 21-81). Most women (76.3%) had FIGO stage I-II disease. Overall, 72.0% had surgery, 70.1% received chemotherapy and 50.7% received radiotherapy. Of 13 patients with known HPV-status, 15% were HPV negative. Median overall survival (OS) was 24 months (range: 0.5-151), with 3- and 5-year OS of 42% and 29%, respectively. To date, the occurrence of SIADH in cervical LCNEC has not been documented, and its presence may serve as an unfavorable prognostic indicator. The survival rate observed in our reported cases was lower than the previously reported median survival rate, potentially attributed to the presence of SIADH among our patients.

Regarding the deficiencies in the treatment of this patient: The clinicians lacked sufficient experience in managing cervical large cell neuroendocrine carcinoma (LCNEC), a rare and highly aggressive tumor. This case was particularly complex due to concurrent pregnancy, syndrome of inappropriate antidiuretic hormone secretion (SIADH) causing refractory hyponatremia, initial pelvic lymph node metastasis, and large tumor volume—features indicative of higher-grade malignancy and more aggressive behavior. Following the initial follow-up, secondary radiotherapy was administered to the localized lymph nodes. However, further investigations such as whole-body PET-CT were not performed in a timely manner. Moreover, systemic therapy including paclitaxel-platinum (TP) chemotherapy, immunotherapy, and targeted therapy was initiated relatively late.

### Treatment of SIADH-induced refractory hyponatremia with tolvaptan

Cervical LCNEC has neuroendocrine functions, and the tumor can directly and ectopically secrete ADH (antidiuretic hormone) or ADH-like substances, or indirectly stimulate the hypothalamus to release excessive ADH. Abnormal ADH secretion can lead to excessive water reabsorption through the “water channels” in the distal convoluted tubule and collecting duct of the kidney, causing hyponatremia and decreased plasma osmotic pressure. The core diagnostic criteria for SIADH must meet all the following conditions: Hyponatremia: Serum sodium <135 mmol/L (typically <130 mmol/L is clinically significant);Hypo-osmolality: Plasma osmolality <275 mOsm/kg (consistent with hyponatremia); inappropriately elevated urine osmolality: urine osmolality >100 mOsm/kg (usually higher than plasma osmolality, indicating inappropriate urine concentration by the kidneys); inappropriately increased urinary sodium excretion: urinary sodium >30 mmol/L (under normal salt intake and euvolemic conditions); normal blood volume (absence of hypovolemia or hypervolemia): no signs of dehydration (e.g., poor skin turgor, hypotension) or edema/ascites (e.g., heart failure, cirrhosis); exclusion of other causes of hyponatremia: normal thyroid function (TSH normal, ruling out hypothyroidism); normal adrenal function (cortisol normal, ruling out adrenal insufficiency); normal renal function (eGFR >60 mL/min, ruling out renal salt wasting or chronic kidney disease). The case we reported underwent relevant tests, revealing:Serum sodium 119 mmol/L (<135 mmol/L), Plasma osmolality 264.0 mOsm/(kg·H_2_O) (<275 mOsm/(kg·H_2_O)), Urine osmolality 400 mOsm/(kg·H_2_O) (>100 mOsm/kg), Urinary sodium 110 mmol/L (>30 mmol/L),24-hour urinary sodium excretion 332 mmol/24h (normal range: 130–260 mmol/24h).The patient was euvolemic (no signs of hypovolemia or hypervolemia), and no diuretics were used during the treatment period. Thyroid ultrasound, adrenal CT, pituitary MRI, and tests for cortisol, adrenocorticotropic hormone (ACTH), and thyroid function showed no significant abnormalities. These findings met the core diagnostic criteria for SIADH, strongly suggesting that SIADH was secondary to LCNEC. The patient was treated with tolvaptan 15 mg/day, maintaining serum sodium levels at 134–145 mmol/L. The dose was gradually reduced by 3.75 mg weekly and discontinued after 4 weeks, and replaced with oral sodium chloride capsules (6 g/day). The patient’s serum sodium levels remained within the normal range, further confirming our diagnosis of SIADH. Hyponatremia, the most common fluid and electrolyte disorder in SIADH, is secondary to various underlying conditions. It can prolong hospitalization, increase healthcare costs and resource utilization, and may even lead to death in severe cases, necessitating prompt treatment. Therefore, timely treatment is extremely important. Bilgetekin I et al. reported the effect of hyponatremia in cancer patients with a SIADH on survival, showing that normal sodium levels after treatment reduced the risk of death (9).Tai P et al. reported that patients with SCLC(small cell lung cancer) complicated by abnormal SIADH had shorter five-year overall survival and worse prognosis. It was shown that serum sodium level was helpful for post-treatment monitoring of SIADH in SCLC patients, and 10% of patients recurred when SIADH occurred. Therefore, SIADH may be a poor prognostic factor for SCLC (10). Tolvaptan is a selective inhibitor of the V2 receptor for ADH that directly inhibits aquaporins in the distal convoluted tubules and the collecting ducts of the kidney, which in turn inhibits water reabsorption and promotes water excretion without affecting Na+ reabsorption. As a result, it can increase Na+ concentrations in plasma. Currently, tolvaptan is mainly used in patients with congestive heart failure and cirrhosis with ascites, and there are very few reports on its use in patients with cervical LCNEC with hyponatremia. In this case, the patient was given tolvaptan tablets, and blood Na+ levels quickly returned to normal. However, side effects of torvaptan include dry mouth, thirst, frequent urination and other symptoms, and its use may also lead to serum sodium overcorrection. When serum sodium concentration rises rapidly, it may result in osmotic demyelination syndrome, which is typically characterized by dysarticulation, silence, dysphagia, lethargy, mood changes, spastic quadriplegia, epilepsy, coma, and death. Although there were no patients with osmotic demyelination syndrome in this study, the incidence of serum sodium overcorrection of tolvaptan was high. Therefore, serum sodium concentration of patients should be closely monitored to prevent the occurrence of osmotic demyelination syndrome caused by overcorrection of serum sodium.

Regarding the deficiencies identified in the management of this patient: During the initial diagnosis, the clinicians demonstrated an insufficient understanding of the patient’s hyponatremia. A comprehensive medical history was not obtained, and management was restricted to symptomatic sodium replacement therapy without pursuing further diagnostic investigations to identify the underlying cause. There was a notable lack of experience in managing hyponatremia secondary to SIADH (Syndrome of Inappropriate Antidiuretic Hormone Secretion). During the initial cycle of chemotherapy, the administration of aggressive fluid therapy inadvertently increased urinary sodium excretion, which subsequently precipitated severe hyponatremia and its associated complications. The therapeutic team lacked experience with the use of tolvaptan for SIADH-induced hyponatremia. In the initial treatment phase, tolvaptan was concurrently administered with both oral and intravenous sodium supplementation. Although this approach did not lead to osmotic demyelination syndrome, it resulted in an overcorrection of the serum sodium concentration.

### Comparative analysis of SIADH in neuroendocrine carcinomas

The Syndrome of Inappropriate Antidiuretic Hormone Secretion (SIADH) is a paraneoplastic condition characterized by hyponatremia, serum hypo-osmolality, and inappropriately concentrated urine due to the ectopic production of arginine vasopressin (AVP) by tumor cells (11). While most commonly associated with small cell lung cancer (SCLC), it is a recognized, though rare, feature of high-grade extrapulmonary neuroendocrine carcinomas (NECs) (11, 12).

### Cervical small cell neuroendocrine carcinoma

Cervical SCNEC is the most frequent high-grade neuroendocrine tumor of the cervix, though it remains a rare entity overall (2, 13). Its association with SIADH is well-established and represents the most common paraneoplastic etiology among cervical NEC types (14, 15). This strong link is attributed to its histopathological and biological similarity to SCLC (16). SIADH in cervical SCNEC typically presents at diagnosis or recurrence, serving as a clinical biomarker for disease activity and burden (14).

### Extrapulmonary neuroendocrine carcinomas (general)

The association of SIADH with extrapulmonary NECs is strongly correlated with tumor grade. High-grade NECs (e.g., of the gastrointestinal tract, bladder, or esophagus) demonstrate a significant propensity for ectopic AVP production, mirroring the behavior of SCLC and cervical SCNEC (11). In contrast, well-differentiated neuroendocrine tumors (NETs) are seldom associated with SIADH and are more frequently linked to other hormonal syndromes (11, 12).

### Cervical large cell neuroendocrine carcinoma

Cervical LCNEC is an exceedingly rare and aggressive malignancy with a poor prognosis (2, 13). The occurrence of SIADH in this specific subtype is exceptionally rare, with only a few documented cases in the literature.

#### Characteristics of SIADH in cervical LCNEC

Diagnostic Presentation: SIADH often serves as the initial presenting symptom, leading to the discovery of the underlying malignancy. Patients present with neurological symptoms of hyponatremia, while cervical symptoms may be absent or non-specific.

#### Clinical biomarker

The syndrome acts as a sensitive surrogate marker of tumor activity. Normalization of serum sodium correlates with treatment response to chemotherapy or radiation, while its recurrence is a highly sensitive indicator of disease relapse, often preceding radiological evidence.

#### Prognostic implication

The development of SIADH is almost invariably associated with advanced, metastatic, or recurrent disease, underscoring the aggressive nature of LCNEC and the critical need to manage the electrolyte imbalance to enable anticancer therapy.

#### Pathophysiology

Despite their “large cell” morphology, these tumors exhibit neuroendocrine differentiation (positive for synaptophysin, chromogranin, CD56) and retain the capacity for ectopic hormone production, including AVP (2).

Pregnancy with cervical LCNEC with SIADH is rare, and hyponatremia may be a predictor of poor prognosis and recurrence. Due to the poor prognosis of cervical LCNEC, more reports and studies are needed to develop the most appropriate treatment for these patients. Due to the low prevalence of this tumor, it may not be possible to establish a standard treatment regimen, and multi-center randomized controlled trials may not be feasible. Case reports and series may be the only means of documenting and accumulating experience for treatment of this disease.

## Data Availability

The original contributions presented in the study are included in the article/supplementary material. Further inquiries can be directed to the corresponding author.
